# Do Patients With Osteoarthritis get Weight Loss Counseling?

**DOI:** 10.7759/cureus.11502

**Published:** 2020-11-16

**Authors:** Syed Hashim Ali Inam, Bismah Riaz, Hamza Jamil, Daneyal Rafique, Umair Asif Siddiqi, Mishal Iqbal, Nawabzada Zeerak Farhat Sherwani, Waqas Khan

**Affiliations:** 1 Internal Medicine, Army Medical College, National University of Medical Sciences (NUMS), Rawalpindi, PAK; 2 Internal Medicine, CMH Lahore Medical and Dental College, Lahore, PAK; 3 Internal Medicine, Military Hospital, Rawalpindi, PAK

**Keywords:** arthritis, weight loss counselling, exercise, physician counselling, osteoarthritis

## Abstract

Introduction: Osteoarthritis (OA) is a degenerative disease of joints which if untreated can lead to a permanent disability of joints. Obesity plays an important role in the morbidity of OA. Since there is no curative treatment for OA, several researches focusing on nonpharmacological intervention for OA have come forth. Triad of education, exercise, and weight loss has been gaining popularity as a first-line nonpharmacological treatment for OA. This article measures the number of OA patients, irrespective of age and gender, who have received weight-loss counseling from their physicians and it also studies patients' willingness to lose weight after being counseled.

Methodology: A cross-sectional study was conducted from 10th June 2020 to 10th July 2020. Diagnosed cases of OA were included and their consent was taken. A self-administered questionnaire was used which included questions asking if they have ever received weight-loss counseling and if they will try to lose weight on being advised by their physician. Data were collected from the participants using google forms and analyzed using SPSS-22. Results: Out of 199 OA patients included in our study, only 28 (14%) participants received weight loss and exercise counseling from their physicians. A positive response was observed in 175 (87.9%) participants out of 199 who reported that they would exercise and practice a healthy lifestyle to lose weight if they were advised properly. Conclusion: Results of our study showed that the total number of OA patients receiving advice from their treating physician regarding weight loss is less. However, the majority of the patients were willing to exercise and control their weight if advised properly by their physician.

## Introduction

Osteoarthritis (OA) is a degenerative joint disorder [1]. It is diagnosed clinically, and radiological evidence supports the diagnosis [2]. According to the World Health Organization (WHO), OA will become the fourth leading cause of disability by the end of the year 2020 [2].

Obesity and being overweight has a strong relation with OA’s progression and its prevalence [3]. In 2015-2016, the prevalence of obesity was 39.8% in adults [4]. The prevalence of obesity is increasing day by day with almost one-third of the world’s population included in the overweight or obese category [5]. According to an estimate, approximately 60% of the world population will be overweight by 2030 if the current trends continue [6].

A directly proportional relation is established between weight loss and symptomatic improvement of OA [7]. Losing weight is considered to be a research-proven first-line therapy for OA [8]. Because of this relationship between weight loss and OA morbidity improvement, the role of exercise and weight loss counseling by the physician is of paramount importance [9]. All international practice guidelines such as Osteoarthritis Research Society International (OARSI) guidelines and the National Institute for Health and Care Excellence (NICE) guidelines recommend that patients with OA should be counseled to lose weight [10-11]. Proper weight loss counseling from the physicians in OA patients is known to improve patient compliance and health outcomes [12]. 

Due to the scarcity of literature on the relationship between OA prognosis and weight-loss counseling, there was limited data to compare our results with. However, our article will add to the literature on this topic. In light of these facts, our study aims to discover the actual number of patients receiving weight-loss advice from their physicians as a part of the treatment of OA.

## Materials and methods

A cross-sectional study was conducted at a tertiary care hospital in Rawalpindi, Pakistan from 10th June 2020 to 10th July 2020. Patients diagnosed with OA who gave consent to participate in the research were included in the study. A self-designed questionnaire was used. Data were collected using google forms after the consent was taken. The confidentiality of the data was maintained. The data were analyzed using SPSS-22. A convenient sampling technique was used and 199 OA patients agreed to participate in the study in this period. The first portion of the questionnaire consisted of patient’s demographics and the second portion had questions asking about the duration of arthritis, counseling received or not, willingness to lose weight after the advice of the doctor, and source of counseling.

## Results

**Figure 1:**
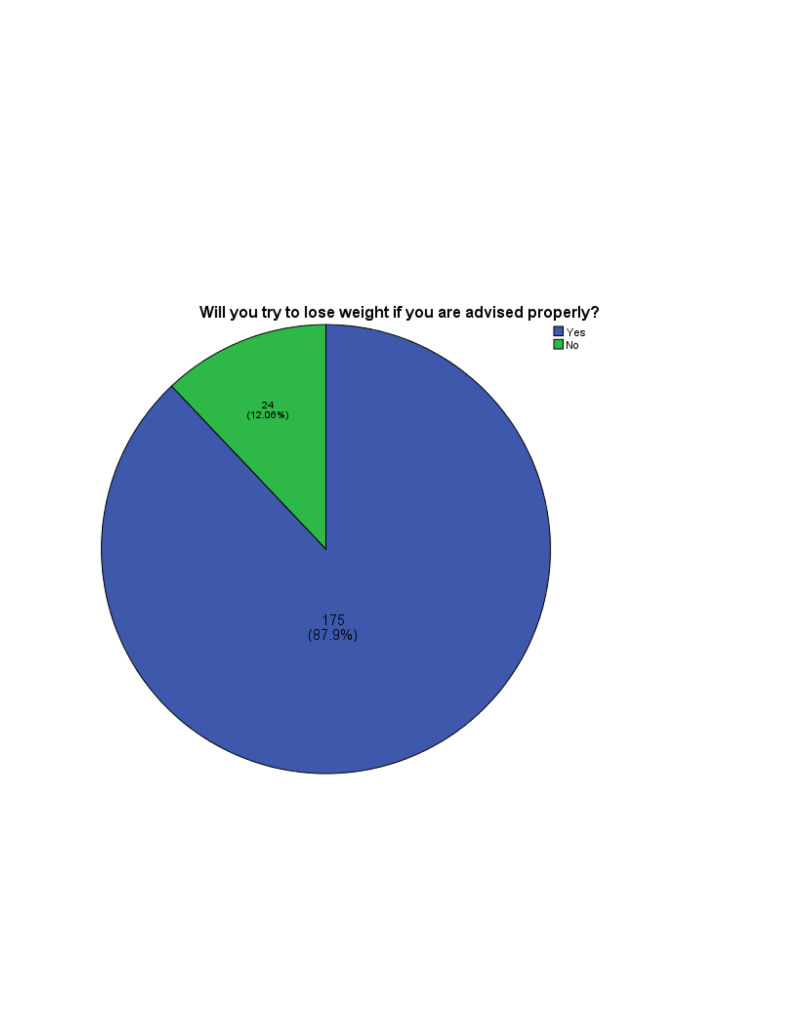
Percentage of OA patients willing to lose weight if advised. OA, osteoarthritis

Our study included a total of 199 participants, out of which 130 (65.3%) were females and 69 (34.7%) were males. On inquiring about the education of patients, 87 (43.7%) had studied till high-school, 35 (17.6%) had completed their bachelor's degree, and 27 (13.6%) had done their master's. Around 50 (25.1%) participants had never been to school and were included in the category of uneducated. A total of 70 (35.2%) participants were diagnosed with OA more than five years ago and 32 (16.1%) had OA for only a year. The duration of the rest of the patients was between one and five years. The mean duration of OA diagnosis was 2.44 years with a standard deviation of 1.011. Among participants, only 28 (14%) out of 199 had received weight-loss counseling from doctors or health care professionals, and 171 (86%) reported that they had not received any advice regarding weight loss from doctors. On inquiring if the participants had ever received information regarding OA management from sources other than doctors, 42 (21%) participants agreed that they acquired knowledge from the internet. If counseled properly, most participants (87.9%) were willing to lose weight with a p-value of 0.01 (alpha ≤0.05), illustrated in Figure 1.

A Chi-square test was applied to see if there was a statistically significant difference between education and the patient's will to lose weight but the results showed a p-value of more than 0.05 for all categories of education. From this, we can conclude that education has no significant relation with a willingness to lose weight, as shown in Table 1.

**Table 1:** Comparison of the level of education with the willingness to lose weight.

Education status	Willing to lose weight	Unwilling to lose weight	p-Value
Uneducated	42	8	0.32
High school	76	11	0.82
Bachelor’s degree	32	3	0.49
Master’s degree	25	2	0.42

.

## Discussion

Lifestyle modification has been gaining increasing recognition as an important part of the management of OA [13]. Emphasis is being made regarding the importance of counseling on increased physical therapy and weight loss in patients with OA [13]. Reduction in body weight has proven to be beneficial for OA patients in several ways [14]. There are researches that support that body fat reduction may improve the prognosis of knee OA more than pharmacologic therapies [15]. NICE guidelines also propose that treatment of OA starts with a nonpharmacological approach which includes exercise [16]. The mechanism by which obesity aggravates OA is not well known [17]. An overweight patient who previously had a normal BMI is at a relatively higher risk of OA than patients who were persistently overweight [14-18].

Our study showed that less than half of the participants had received weight-loss counseling. According to a study, overweight and obese patients with OA were more likely to lose weight if advised adequately [17, 19-20]. Clinicians have been facing several difficulties in counseling OA patients regarding weight loss [21]. These difficulties being: insufficient time during an appointment, lack of awareness of appropriate exercises, and uncertain billing procedures [21-22]. Some adults with OA have reported being unwilling to exercise due to their physical limitations. They feel that their activity is limited due to pain in their knees, increasing age, and other diseases if present. Therefore, interventions using cognitive behavioral therapy can lead to significant weight loss [23-24]. Physicians and other healthcare professionals should not underestimate the influence of their advice on the behavior of patients. Many physicians have been reported to consider OA less important than other life-threatening conditions so less time and work is being put towards its management and research [25-26].

In our study, the majority of participants were willing to lose weight after they have received weight-loss counseling (p-value ≤ 0.05). On studying the relation between weight-loss counseling and the education level of participants, we concluded that education has no significant effect on the willingness to lose weight after counseling. From this, we can deduce that willingness to lose weight is mostly related to whether the counseling has been received or not, and not related to the education level of the participants. 

Our study has been limited by the fact that all data on weight loss counseling are based on self-reports. Therefore, recall bias is possible. Another limitation is that the data were collected from a single tertiary care center on a limited number of participants so the study results cannot be generalized to a bigger population.

Further research is required to emphasize the importance of weight loss in the management of OA. Steps should be taken by health authorities to educate clinicians to incorporate weight-loss counseling in their practice. Moreover, health awareness schemes regarding the effect of weight loss on OA prognosis should also be made possible.

## Conclusions

As per our study, the number of OA patients who receive weight-loss counseling is low. Physicians need to counsel patients because as per our research, a significant number of OA patients were willing to lose weight if they have been counseled (p value ≤ 0.05). Our study also showed that willingness to lose weight is mostly related to whether the counseling has been received or not, and not related to the education level of the participants. Further studies are needed to understand the obstacles healthcare professionals are facing with weight-loss counseling and weight-loss counseling effects on the behavior of patients. Steps should be taken to encourage healthcare professionals to increase the counseling frequency regarding weight loss in OA patients.
